# Set Size of Information in Long-Term Memory Similarly Modulates Retrieval Dynamics in Young and Older Adults

**DOI:** 10.3389/fpsyg.2022.817929

**Published:** 2022-03-02

**Authors:** Jan O. Peters, Tineke K. Steiger, Alexandra Sobczak, Nico Bunzeck

**Affiliations:** ^1^Department of Psychology, University of Lübeck, Lübeck, Germany; ^2^Center of Brain, Behavior and Metabolism (CBBM), University of Lübeck, Lübeck, Germany

**Keywords:** recognition memory, set size, aging, top-down, bottom-up

## Abstract

Our ability to rapidly distinguish new from already stored (old) information is important for behavior and decision making, but the underlying processes remain unclear. Here, we tested the hypothesis that contextual cues lead to a preselection of information and, therefore, faster recognition. Specifically, on the basis of previous modeling work, we hypothesized that recognition time depends on the amount of relevant content stored in long-term memory, i.e., set size, and we explored possible age-related changes of this relationship in older humans. In our paradigm, subjects learned by heart four different word lists (24, 48, 72, and 96 words) written in different colors (green, red, orange, and blue). On the day of testing, a color cue (e.g., green) indicated with a probability of 50% that a subsequent word might be from the corresponding list or from a list of new words. The old/new status of the word had to be distinguished *via* button press. As a main finding, we can show in a sample of *n* = 49 subjects, including 26 younger and 23 older humans, that response times increased linearly and logarithmically as a function of set size in both age groups. Conversely, corrected hit rates decreased as a function of set size with no statistically significant differences between both age groups. As such, our findings provide empirical evidence that contextual information can lead to a preselection of relevant information stored in long-term memory to promote efficient recognition, possibly by cyclical top-down and bottom-up processing.

## Introduction

Efficient recognition of familiar information cannot be based on a sampling of all information stored in long-term memory but it is supposed to be modulated by top-down contextual information. For instance, finding a friend in a crowd of unknown people should be faster if it is clear which specific person to search for. Therefore, retrieval dynamics should be related to the number of relevant items stored in long-term memory (i.e., memory load, set size, or list length). Indeed, in recognition memory tasks, response time (RT, i.e., retrieval speed) to old and new items is fast when the list of initially encoded items is short, and it increases with list length (Ross, 1970; Burrows and Okada, 1975). While this is further supported by semantic priming studies (Graf and Mandler, 1984), the specific relationship between set size and RT is less clear. In Sternberg’s, (1966) initial work on working memory, a linear relationship has been interpreted as a serial process. Subsequent analyses, however, also provide evidence for a logarithmic fit, pointing toward parallel computations in both working (Brigg, 1974) and long-term memory tasks (Ross, 1970). Some studies even reported both a bilinear and logarithmic relationship (Burrows and Okada, 1975), which could be indicative of differential processes underling recognition of information from working and long-term memory.

Prominent evidence for logarithmical scaling comes from modeling work by Graboi and Lisman (2003) suggesting that recognition memory is based on a bidirectional flow of information, including the interplay between bottom-up and top-down processes. In general terms, bottom-up is the flow of information from lower to higher regions or processing modules (e.g., receptors to the cortex). It mainly relies on sensory information. Top-down processing, on the other hand, describes the flow of information from higher to lower regions or processing modules, and therefore, relies on prior knowledge and experience (see, e.g., Rauss and Pourtois, 2013). More specifically, the model by Graboi and Lisman (2003) implies that top-town information, such as a contextual cue, activates a set of relevant information (nodes) that guides further sampling during the recognition process. Specifically, when subjects have to recognize a visually presented word from a previously learned list, attention is being guided to information-rich features, for instance a rare letter with a low probability to be contained in any of the learned words. Subsequently, all words from the previously learned list will be excluded if the feature is not detected, which leads to a re-computation of top-down feature probabilities, that guides further information processing. Since a constant portion of words (e.g., half the words) will be excluded during each of these cycles, and since our ability to move covert attention is limited to 20–30 ms (Horowitz and Wolfe, 1998), the recognition process is supposed to scale logarithmically with set size (Graboi and Lisman, 2003).

While a few more recent studies support a relationship between set size and recognition dynamics (Wolfe, 2012; Drew and Wolfe, 2014), they have neglected potential age-related changes. Episodic memory (i.e., personal experiences with temporal and spatial information) is particularly affected during healthy aging (Tromp et al., 2015). Semantic memory and memory for individual items, on the other hand, are often preserved until old age (Hedden and Gabrieli, 2004; Saverino et al., 2016). One possible explanation for such dichotomy is that episodic and semantic memory relate to common but also specific underlying neural brain regions that show different age-related neural degenerations. For instance, the prefrontal cortex and medial temporal lobe show pronounced age-related neural degenerations and play a role in episodic and associative memory (Hedden and Gabrieli, 2004). Along these lines, studies in amnesic patients, who have difficulties in recalling information, show intact semantic priming in a word-completion test (Graf et al., 1985). In contrast, patients with Alzheimer’s disease, but not healthy older subjects, may be impaired in the word-completion test (Fleischman and Gabrieli, 1998; Millet et al., 2010; De Wit et al., 2021).

In this study, we investigated the relationship between set size of information stored in long-term memory and retrieval dynamics in a recognition task. To this end, we conceived a novel paradigm in which subjects learned four different word lists (24, 48, 72, and 96 words) in different colors over several days. At retrieval, a colored cue indicated the appearance of a word from the associated word list or a novel word (not presented in any of the word lists), and subjects had to indicate the old/new status of the word *via* button press. On the basis of the work by Graboi and Lisman (2003), we expected a logarithmic relationship between set size and RTs in both young and older subjects. We also expected worse overall memory in older subjects given that the task involves associative learning, and we explored possible age-related differences in the relationship between set size and RTs.

## Materials and Methods

### Participants

To be included, subjects had to be right-handed, currently healthy with no history of neurological or psychiatric disorders (self-report) and German as native language. All participants had normal or corrected-to-normal vision (including color vision), and they were not taking any medication at the time. Together, 29 younger and 28 older subjects participated in our study. One older subject was excluded due to low memory scores in the list learning task (corrected hit rate <0.1) and three were excluded due to a hit rate of <0.25 in the color-assignment task (i.e., below chance, see below). Additionally, one older subject was excluded due to a score of 21 in the Montreal Cognitive Assessment (MoCA; see below). One younger subject was excluded due to low memory scores in the list learning task (corrected hit rate <0.1), one was excluded due to a hit rate of <0.25 in the color-assignment task (i.e., below chance, see below), and one was excluded due to outlying values in hit RTs in the recognition task (>3 SD above the group mean). Therefore, a sample of *n* = 26 younger (seven male, mean age = 23.46 years, *SD* = 4.19 years, range: 19–36 years) and *n* = 23 older (seven male, mean = 63.61 years, *SD* = 7.43 years, range: 51–75 years) subjects remained and was analyzed. This age range was chosen since the medial temporal lobe, which is particularly involved in declarative long-term memory functions, typically degenerates with age, starting at around 50 years (Raz et al., 2004). Since we used a novel paradigm, we could not perform a literature-based *a priori* estimation of power and sample size.

Participants were recruited through announcements in the local newspaper or a database of the University of Lübeck (Greiner, 2015). While the former was a brief paragraph describing the study, the latter was delivered *via* e-mail. If interested, potential candidates got in contact *via* phone or e-mail to receive further information. On the day of testing, all signed a written informed consent and, after completion, received monetary compensation or study course credits. While most younger participants were university students, older participants were recruited from a much more diverse range of environments (see discussion for potential caveats). Before any recruitments, the study was approved by the local ethical committee of the University of Lübeck, Germany, and in accordance with the Declaration of Helsinki.

### Study Design

The study contained two parts. In a first meeting, subjects were instructed to learn words (i.e., encoding a study list, see below), and in a second meeting, memory for the words on the study list was tested using a recognition memory paradigm (i.e., retrieval, see below). The second part (retrieval) took place 5 to 6 days after the first meeting. After completing the retrieval part, all subjects filled out a short questionnaire in which they were asked about their learning experience. It revealed that learning the word lists started on average 3.9 days (younger subjects) or 4.3 days (older subjects) before the recognition memory test. Additionally, the older subjects were tested with the Geriatric Depression Scale questionnaire (GDS; Yesavage et al., 1982) and MoCA (Nasreddine et al., 2005). All subjects had GDS values of <6 (indicating no depression). As alluded to above, one older subject had to be excluded due to a MoCA value of 21 indicating mild cognitive impairment (Freitas et al., 2013). Finally, the subjects were fully informed about the content and purpose of the study in a debriefing. Altogether, both parts took about 3 h. For quality criteria, including validity and reliability, of the GDS and MoCA, see, e.g., Yesavage et al. (1982) and Nasreddine et al. (2005).

The study list contained four different word lists with different length (24, 48, 72, and 96 words; in sum: 240 words) written in different colors (red, blue, green, and orange). All words were taken from the Berlin Affective Word List-Reloaded (Võ et al., 2009), a database with more than 2,900 German words. All words were five to eight letters long and had neutral ratings with regard to emotional valance, arousal, and imageability (e.g., paddle, folder, and frog). To avoid effects of word order, position or color, the word to set size association, and set size to color associations were randomly permuted, resulting in the four different study lists A, B, C, and D (each including four colored word lists with different length). Note that only one study list was given to each subject.

During the first meeting, subjects were instructed to learn the words by heart but to avoid any specific learning strategies, such as organizing words into living/non-living. However, they were free in their learning duration and frequency, and they were instructed that a memory test on the words would be performed during the second meeting.

**Figure 1:**
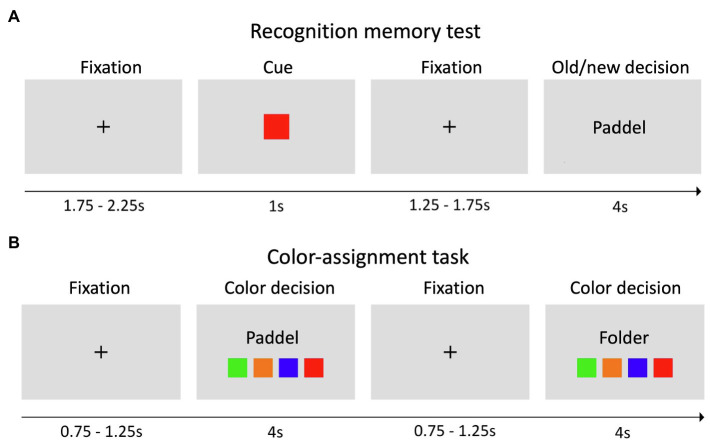
Experimental paradigm. **(A)** In the recognition memory test, each trial started with a fixation cross, followed by a colored cue, a fixation cross, and an old or new word. Old words were always from the corresponding color list, as indicated by the cue, and participants were asked to consider this information to solve the task. For each presented word, subjects had 4 s to indicate whether they recognized the word (old) or not (new) *via* button press. **(B)** In the color-assignment task, subjects were presented only with old words and had to identify the color list, from which the word was drawn.

In the second meeting, all participants performed a recognition memory task with a total of 192 words, including 96 words from the study list and 96 novel words (i.e., words not included in any word list; Figure 1A). Specifically, for the old words, we randomly chose 24 words from each of the four word lists and randomly presented them together with new words in four blocks of 48 trials. Every trial started with a fixation cross, followed by a color cue (e.g., green) indicating that a subsequent word might be presented from the corresponding list or from a list of new words. This was followed by a fixation cross and the corresponding old or new word (50% probability). Within 4 s, an old/new distinction had to be made. While “old” indicated that the word was recognized from the previously learned word list, “new” indicated that the word was not on the list and therefore not recognized. Importantly, all subjects were instructed about the task structure and to take the color information into account. Before the beginning of the task, all subjects performed a brief training session.

To assess if color-word associations had been correctly learned, an additional color-assignment task was implemented. We randomly presented 24 different old words (six from each word list) together with four different squares corresponding to the study list (Figure 1B). The subjects were instructed to indicate the color of the list associated with the word *via* button press. In both the recognition memory task and the color-assignment task, words were written in black font and subjects were instructed to respond as quickly and accurately as possible.

During the second meeting (i.e., recognition), brain activity from the younger subjects was measured using electroencephalography. These data will be reported elsewhere.

### Data Analysis

Behavioral data were analyzed using Matlab,^1^ SPSS,^2^ and Jamovi^3^ (including the Plugin JSQ developed by the JASP Team). For the recognition memory test, we assessed recognition memory performance based on corrected hit rates, which were calculated by subtracting the proportion of false alarms (incorrect old responses to new words) from the proportion of hits (correct old responses to old words) for each word list. We also analyzed RTs during recognition for each of the four word lists and removed trials faster than 250 ms (Woods et al., 2015), or trials slower than three SD above the mean RT.

Since many of the data did not meet the necessary conditions (normal distribution, homoscedasticity, sphericity; Shapiro–Wilk-Test <0.05; Levene-Test <0.05; and Mauchly-Test <0.05), we used permutation tests with 10.000 random samples, including the inner subject factor set size and between subject factor age group. Main effects and interactions were tested using F-scores, and *post-hoc* comparisons were tested using t-scores. Statistical significance was assessed on the basis of Monte Carlo values of *p* (North et al., 2002). The relationship between set size and RT was further tested using linear and logarithmic fits. To better interpret our findings, especially non-significant effects, we additionally performed Bayesian ANOVAs and *post-hoc t*-tests (Schönbrodt and Wagenmakers, 2018). Specifically, four models were tested against the null model, including: (1) set size, (2) age group, (3) both set size and age group, and (4) set size, age group, and the interaction set size x age group.

While values of *p* only indicate whether a null hypothesis can be rejected, the Bayes factor indicates the evidence in favor of the alternative vs. the null hypothesis given the empirical data. Bayes factors close or equal to one suggest inconclusive evidence, i.e., they neither demonstrate evidence for H1 nor for H0. Bayes factors of 1–3 (for H1) and 1–1/3 (for H0) indicate “anecdotal evidence”; Bayes factors of 3–10 (for H1) and 1/3–1/10 (for H0) indicate “moderate evidence”; Bayes factors of 10–30 (for H1) and 1/10–1/30 (for H0) indicate “strong evidence”; Bayes factors of 30–100 (for H1) and 1/30–1/100 (for H0) indicate “very strong evidence”; and Bayes factors of >100 (for H1) and <1/100 (for H0) indicate “extreme evidence” (Schönbrodt and Wagenmakers, 2018). Prior odds and alpha levels (*p* < 0.05) were corrected for multiple comparisons, when appropriate (see Results).

## Results

### Recognition Memory Task

**Figure 2:**
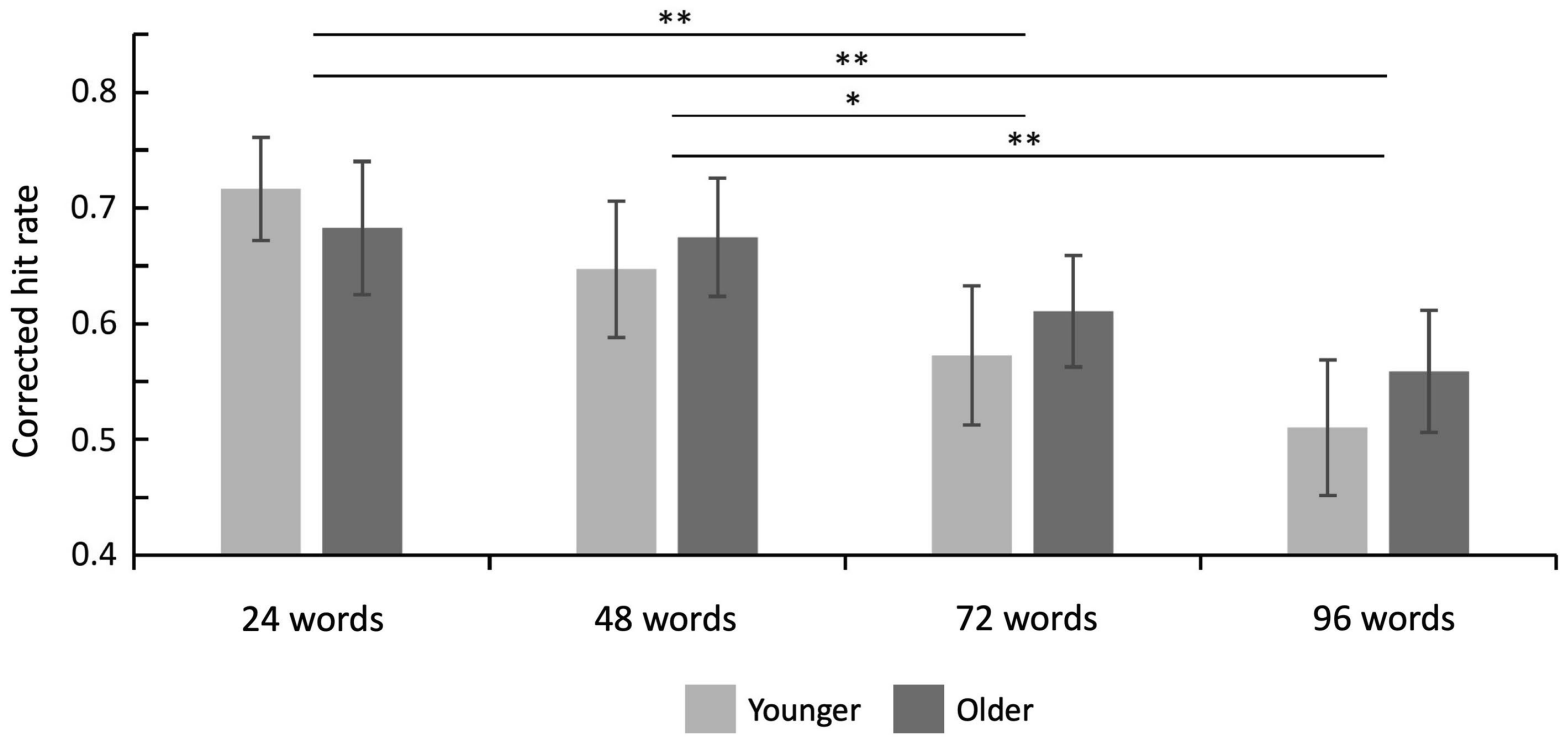
Recognition memory performance. Corrected hit rates decreased with increasing set size (main effect of set size) but this effect did not differ between both age groups. Error bars denote one standard error of the mean; ^*^ < 0.0083; and ^**^ < 0.001.

Hit rates, false alarm rates, and corrected hit rates are shown in Table 1. A 2 × 4 ANOVA with the factors age (younger and older subjects) and set size (24, 48, 72, and 96 words) on corrected hit rates revealed a mean effect of set size [*F*(3,141) = 14.20; *p* < 0.001; ${\mathrm{eta}}_{p}^{2}$ = 0.232], but no significant main effect of age [*F*(1,47) = 0.08; *p* = 0.776; ${\mathrm{eta}}_{p}^{2}$ = 0.002] and no significant interaction [*F*(3,141) = 0.90; *p* = 0.444; ${\mathrm{eta}}_{p}^{2}$ = 0.019]. Six *post-hoc t*-tests on corrected hit rates across both age groups (*α* = 0.0083, Bonferroni correction) revealed significant differences between 24 vs. 72 words [*t*(48) = 3.86; *p* < 0.001; *d* = 0.551], 24 vs. 96 words [*t*(48) = 4.93; *p* < 0.001; *d* = 0.705], 48 vs. 72 words [*t*(48) = 3.22; *p* = 0.001; *d* = 0.460], and 48 vs. 96 words [t(48) = 4.62; *p* < 0.001; *d* = 0.660], see Figure 2. The other two comparisons were not statistically significant.

**Table 1:** Accuracy for the recognition memory task.

Set size	Hit rates		False alarm rates		Corrected hit rates	
	Younger	Older	Younger	Older	Younger	Older
24 words	0.88 (0.02)	0.85 (0.03)	0.16 (0.03)	0.17 (0.04)	0.72 (0.04)	0.68 (0.06)
48 words	0.87 (0.03)	0.85 (0.04)	0.22 (0.04)	0.17 (0.03)	0.65 (0.06)	0.67 (0.05)
72 words	0.81 (0.03)	0.83 (0.03)	0.24 (0.04)	0.22 (0.03)	0.57 (0.06)	0.61 (0.05)
96 words	0.78 (0.03)	0.78 (0.03)	0.27 (0.04)	0.22 (0.04)	0.51 (0.06)	0.56 (0.05)

*Listed are hit rates, false alarm rates, and corrected hit rates. Numbers show mean values and standard errors (in brackets)*.

Bayesian statistics on corrected hit rates showed that, compared to the null model, a model including the factor set size was superior (BF_10_ = 538,394.95), followed by a model including both factors set size and age group (BF_10_ = 242,674.58). The direct comparison of both models showed that the data are more likely to be explained by the model only including the factor set size (BF_12_ = 538,394.25/242,674.58 = 2.22). This was further confirmed by effect analyses showing that set size is the only factor with extreme evidence (BF_Incl_ = 554,218.24). There was moderate evidence against the interaction term of both factors (set size × age group; BF_Incl_ = 0.15), and anecdotal evidence against the factor age (BF_Incl_ = 0.45). *Post-hoc* comparisons were based on data averaged across both age groups, and the Bayes factor was corrected with prior odds of 0.41 [due to multiple comparisons, de Jong (2019)]. They revealed evidence in favor of differences between 24 vs. 72 words (BF_10, U_ = 75.95, posterior odds = 31.46), 24 vs. 96 words (BF_10, U_ = 1907.12, posterior odds = 789.96), 48 vs. 72 words (BF_10,U_ = 13.61, posterior odds = 5.64), and 48 vs. 96 words (BF_10,U_ = 722.24, posterior odds = 299.16). Together, Bayesian statistics confirm the frequentist approach by showing that set size, but not age group or the interaction, mainly explains the observed data.

**Figure 3:**
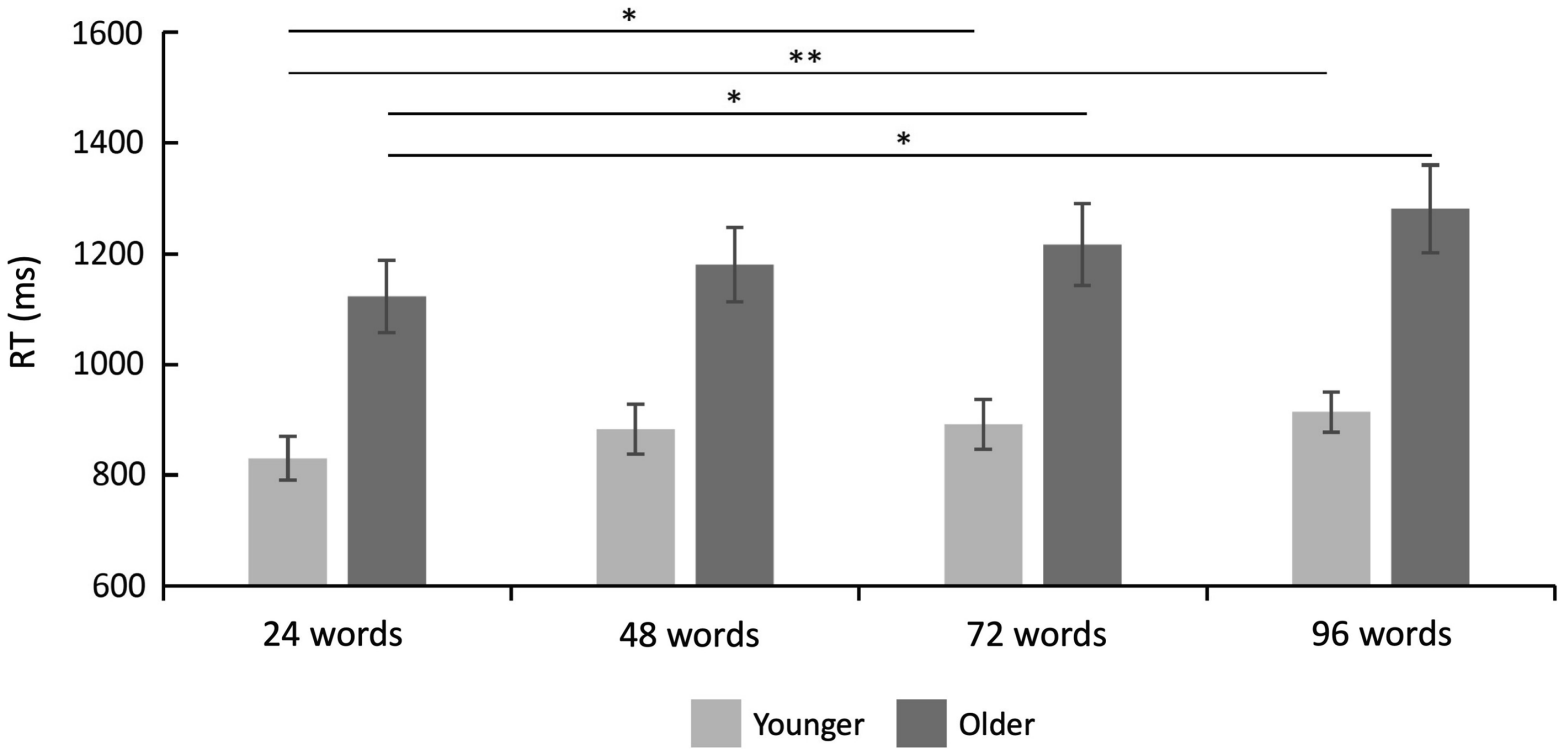
Reaction time results. Reaction times were faster in younger as compared to older adults (main effect of age), and they increased with set size (main effect of set size). There was no significant interaction between set size and age group (see text). Error bars denote one standard error of the mean; ^*^ < 0.0041; and ^**^ < 0.001.

Reaction times are shown in Table 2 and Figure 3. A 2 × 4 ANOVA with the factors age and set size revealed a significant main effect of age [*F*(1,47) = 17.87; *p* < 0.001; ${\mathrm{eta}}_{p}^{2}$ = 0.275], which was driven by faster RTs in the group of younger subjects (mean RT = 879.80 ms ± 41.51) as compared to older subjects (mean RT = 1200.29 ms ± 71.44). It also revealed a main effect of set size [*F*(3,141) = 10.21; *p* < 0.001; ${\mathrm{eta}}_{p}^{2}$ = 0.178], which was—as expected—driven by slower RTs for words from longer learning lists (Figure 3). There was no statistically significant interaction between age and set size [*F*(3,141) = 1.18; *p* = 0.322; ${\mathrm{eta}}_{p}^{2}$ = 0.024] indicating similar effects of set size on RTs in both age groups.

**Table 2:** Reaction times for the recognition memory task.

Set size	Reaction times	
	Younger	Older
24 words	830.39 (39.64)	1122.93 (64.91)
48 words	882.95 (44.92)	1180.43 (67.18)
72 words	891.85 (45.23)	1216.73 (74.02)
96 words	914.01 (36.25)	1281.06 (79.43)

*Listed are mean response times (in ms) and standard errors (in brackets) for the different conditions (set sizes) and groups (younger and older subjects)*.

Twelve *post-hoc t*-tests (*α* = 0.0041, Bonferroni correction) separately for each age group (despite no significant interaction but to fully characterize our data and to fully explore possible age-related differences in the set size effect) revealed, in the group of younger subjects, a significant difference between 24 vs. 72 words [*t*(25) = −3.10; *p* = 0.001; *d* = −0.608] and 24 vs. 96 words [*t*(25) = −4.12; *p* < 0.001; *d* = −0.808]. In the group of older subjects, there was a significant difference between 24 vs. 72 words [*t*(22) = −2.83; *p* = 0.003; *d* = −0.590] and 24 vs. 96 words [*t*(22) = −3.56; *p* = 0.001; *d* = −0.742], see Figure 3. All other *post-hoc* comparisons were not statistically significant.

Bayesian statistics on RTs during recognition showed that, compared to the null model, a model including both factors (set size and age group) was superior (BF_10_ = 369,205.81), followed by a model including both factors and their interaction term (BF_10_ = 76,558.50). The direct comparison of both models showed that the data are more likely to be explained by the model only including both factors set size and age group but not their interaction (BF_12_ = 369,205.81/76,558.50 = 4.82). This was further confirmed by effect analyses showing that both set size (BF_Incl_ = 2498.31) and age group (BF_Incl_ = 135.58) are factors with extreme evidence. There was moderate evidence against the interaction term (BF_Incl_ = 0.21).

*Post-hoc* comparisons were based on separate data for both groups, and the Bayes factor was corrected with prior odds of 0.26 [due to multiple comparisons, de Jong (2019)]. For the younger subjects, they revealed evidence in favor of differences between 24 vs. 96 words (BF_10, U_ = 83.89; posterior odds = 21.81) and evidence for no difference between 48 vs. 72 words (BF_10, U_ = 0.22; posterior odds = 0.06). For the older subjects, *post-hoc* comparisons revealed evidence in favor of differences between 24 vs. 96 words (BF_10, U_ = 21.71; posterior Odds = 5.64). Finally, the RT difference for 96 vs. 24 words was compared between both groups. It revealed no conclusive evidence (BF_10_ = 1.45). Together, the Bayesian statistic confirms the frequentist approach by showing that set size and group but not the interaction mainly explains the observed data.

**Figure 4:**
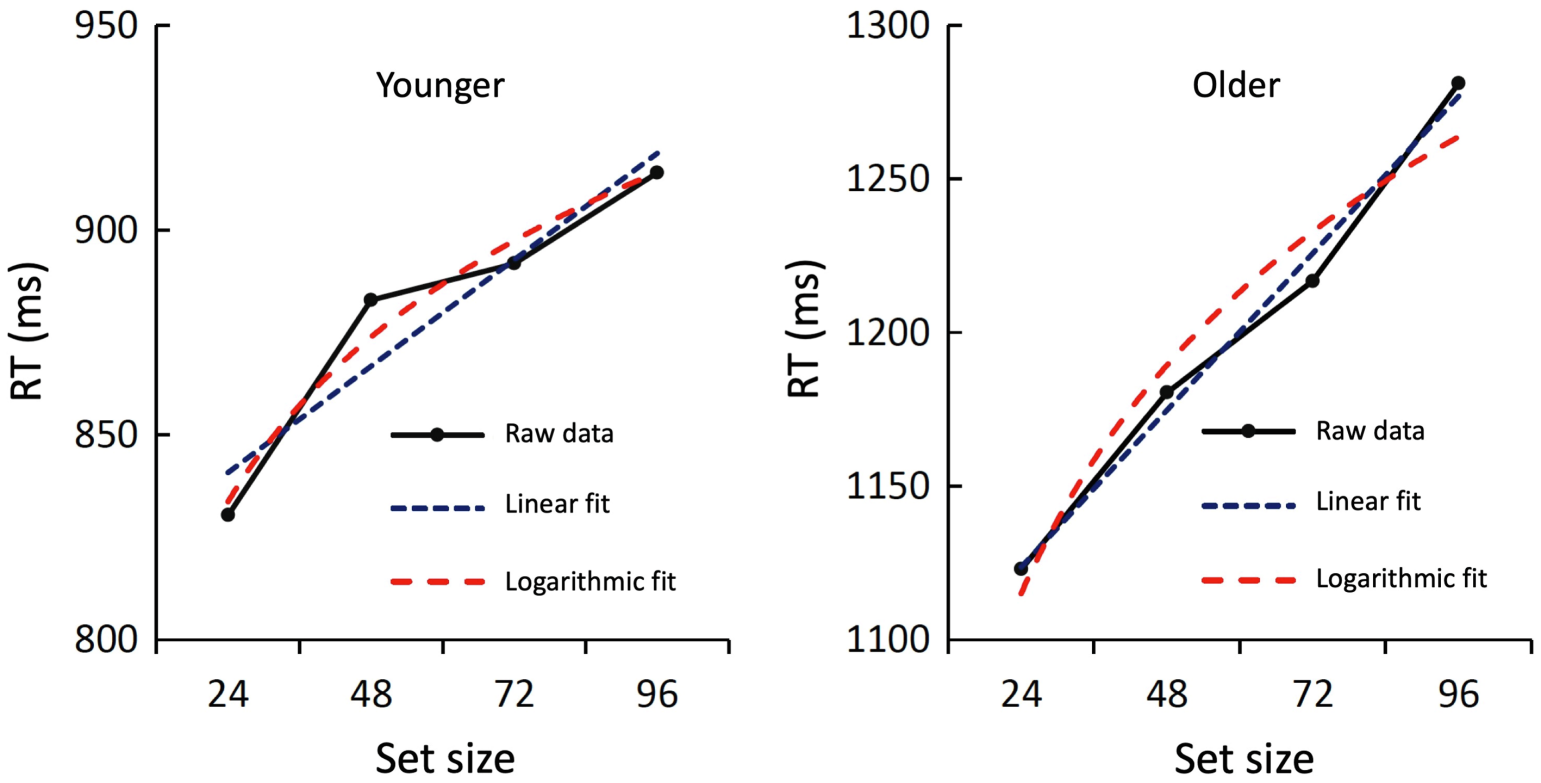
Raw data and regression curves of the RTs during recognition. The averaged raw data of the younger (**left**) and older subjects (**right**) are shown in black, the linear regression curve in blue, and the logarithmic regression curve in red. Both fits were statistically significant or marginally significant (see text).

Finally, to further investigate the relationship between set size and RT, we tested a linear and logarithmic fit for both age groups. For the younger subjects, the linear fit was borderline significant [*F*(1,2) = 17.17; *p* = 0.054; *R*^2^ = 0.896] and the logarithmic fit was significant [*F*(1,2) = 58.83; *p* = 0.017; *R*^2^ = 0.967]. Both curves showed a close fit to the original data (Figure 4). For the older group, both the linear fit [*F*(1,2) = 196.33; *p* = 0.005; *R*^2^ = 0.990] and the logarithmic fit [*F*(1,2) = 35.46; *p* = 0.027; *R*^2^ = 0.947] were statistically significant. Again, both curves showed a close fit to the original data (Figure 4).

### Color-Assignment Task

The main goal of the color-assignment task was to ensure that our participants not only learned the words but also the word-color association. As shown in Table 3, such a conclusion can be drawn since the hit rates were all above chance (*p* = 0.25). Apart from that, we had no specific hypotheses regarding age differences or other effects. Together with the fact that only six trials per condition were included, we did not further analyze these data using inference statistics. For the sake of completeness, we report the hit rates together with the RTs in Table 3.

**Table 3:** Accuracy and RTs for the color-assignment task.

Set size	Hit rates		Reaction times	
	Younger	Older	Younger	Older
24 words	0.86 (0.04)	0.68 (0.06)	1291.40 (91.53)	1999.86 (163.40)
48 words	0.72 (0.06)	0.54 (0.06)	1460.24 (88.65)	2256.01 (157.50)
72 words	0.68 (0.05)	0.51 (0.06)	1553.13 (101.41)	2437.43 (220.32)
96 words	0.74 (0.04)	0.47 (0.05)	1391.10 (68.83)	2067.11 (157.46)

*Numbers show mean hit rates, RTs (in ms), and standard errors (in brackets)*.

## Discussion

Retrieval accuracy and decision speed in an old/new task decreased with set size, and this effect was similarly observed in a group of young and older subjects suggesting that the underlying processes are unimpaired during healthy aging. Importantly, the relationship between retrieval speed and set size followed a linear and logarithmic function providing support for the notion that visual recognition from long-term memory is based on both serial and parallel sampling of information that is guided by bottom-up and top-down processing.

Modeling work (Graboi and Lisman, 2003) suggests that both bottom-up and top-down processes modulate recognition memory. Such a conclusion was also based on empirical work, mainly including working memory paradigms, exploring the relationship between the sampled set sizes of information and RTs. While initial studies (Sternberg, 1966) showed a linear relationship—and therefore point toward a serial process—subsequent meta-analyses also provide evidence in favor of a logarithmic fit, pointing toward parallel computations when retrieving information (Brigg, 1974). This appears to be particularly pronounced when stimulus material is complex (e.g., when including numbers and letters; Brigg, 1974). A similar conclusion was drawn by Ross (1970) with regard to long-term memory, who could show—in a sample of four subjects—a logarithmic set size effect for word-letter combinations even after 20 days.

These seemingly divergent observations (linear vs. logarithmic relationship) were brought together in a study with five subjects who learned lists of two to 20 words, and, in the immediately following recognition test had to decide whether a presented word was included in the previously learned list or not (Burrows and Okada, 1975). Interestingly, the observed set size effect was both bilinear and logarithmic. The bilinear effect, with a separation point at around six to eight words, indicates a serial retrieval process, which appears to differ between short- and long-term memory. The logarithmic effect, on the other hand, indicates a parallel process that is common for both short- and long-term memory (Graboi and Lisman, 2003). Further evidence for parallel processing during retrieval from long-term memory comes from more recent work, again showing a logarithmic relationship between RTs and object lists of two, four, eight, 16, and 100 objects (Wolfe, 2012), see also Drew and Wolfe (2014). Similar to the model by Graboi and Lisman (2003), this effect was interpreted on the basis of a step-wise exclusion of half the objects per cycle (see next paragraph).

How does both serial and parallel computing drive recognition? Top-down information—in our case the colored cue—pre-activates a set of relevant information (nodes) stored in long-term memory that guides further sampling (Graboi and Lisman, 2003). Therefore, information that is contextually irrelevant for the recognition process is being excluded in a first step. During subsequent visual presentation of the old/new word (i.e., bottom-up processing), attention is being guided to “information-rich features,” for instance a letter with low probability, and all words from the list stored in memory are being excluded if this feature is not included. This will lead to a re-computation of top-down feature probabilities that guides further information processing. In each cycle (or bidirectional flow of information), a constant portion of the words will be excluded, and this will be repeated until only one word remains (leading to an “old” decision”)—in the case of a “new” word, the cycles are being repeated until no word remains (leading to a “new” decision). Apparently, the longer the word lists, the more cycles will be computed leading to longer recognition. Specifically, the number of cycles and associated recognition are determined by the set size and the ability of covert attention to be moved every 20–30 ms (Horowitz and Wolfe, 1998). While this suggestion fits to previous EEG studies with cortical old/new signals typical at around 200 ms after stimulus onset (Rugg and Curran, 2007), it remains to be investigated whether this physiological signature also varies as a function of set size. Along the same lines, attentional processes, which are known to be closely related to gamma band oscillations (30–80 Hz; Engel et al., 2001; Fries, 2015), might also directly relate to top-down/bottom-up cycles leading to recognition (Graboi and Lisman, 2003).

On average, older subjects were slower during the old/new recognition task, which was expected based on previous studies (Salthouse, 2000, 2010; Woods et al., 2015; Alejandro et al., 2021). This observation can be accounted for by several aspects. First, the old/new task required a fast motor response which, due to motor impairments during aging, most likely contributed to RTs differences between both age groups (Woods et al., 2015). Second, healthy aging is typically associated with a general slowing in processing speed that is more pronounced in complex reaction tasks (Salthouse, 2000). Along these lines, a more specific slowing in visual processing speed may also have contributed (Ebaid et al., 2017). However, age-related deficits in visual processing seem to be less pronounced in highly familiar tasks (e.g., when reading individual words; Ebaid and Crewther, 2019). Together, slower RTs in our older subjects appear to be based on impairments in motor components and general processing speed.

Both age groups had similar recognition memory performance and—importantly—exhibited a logarithmic relationship between set size and RTs. While age-related differences could have been expected on the basis of well-described memory and attention deficits during aging, it is also clear that recognition and semantic memory are often preserved until old age (Hedden and Gabrieli, 2004; Ofen and Shing, 2013). However, it remains unclear how similar behavioral performance (corrected hit rates in particular) was achieved in this specific task. One possibility is that compensatory processes account for structural neural degeneration (Cabeza et al., 2018), which might be indicated by slower RTs in older adults (see previous paragraph). In other words, a change in speed-accuracy tradeoff could be the basis for equal performance. Alternatively, but not mutually exclusive, older subjects may have used different learning strategies and longer learning times. While older participants started to learn slightly earlier (4.3 vs. 3.9 days before testing) and reported to have investigated a large amount of time, we did not quantify the latter. Similarly, we did not formally ask for any learning strategies, but other studies suggest when learning is self-directed, age-related differences can be smaller (Verhaeghen et al., 1993) and errors reduced (Canestrari, 1963; Kinjo, 2010). Along these lines, it might be helpful to consider the subjects’ level of education or socioeconomic status, which may have differed between groups. In future studies, these aspects could be investigated with appropriate questionnaires and neural compensation with imaging techniques, such as EEG and fMRI.

Results from the color-assignment task show that both age groups performed above chance and therefore not only learned the word lists but also word-color associations. In contrast to the set size recognition memory task (Table 1 and Figure 2), accuracy in the color-assignment task was lower in the older group for all four set sizes suggesting worse associative declarative memory (see Table 3). However, due to the low number of trials per condition (six), no formal inference statics could be performed, and therefore, such conclusions need to be drawn carefully.

Apart from model-based considerations (Graboi and Lisman, 2003), the observed set size effect could have other explanations. For instance, motivation is one of the driving forces behind learning success (Wittmann et al., 2005; Adcock et al., 2006; Gruber et al., 2014) and longer word lists may have been associated with less motivation or attentional resources (Kinnell and Dennis, 2011). Therefore, shorter word lists could have been repeated more often leading to better memory and faster retrieval. Indeed, repeated retrieval of information not only promotes recognition memory performance— so-called testing effect—(Karpicke and Roediger, 2008; Karpicke and Blunt, 2011; Guran et al., 2019, 2020), but it also accelerated RTs during retrieval (Ross, 1970; Dosher, 1984). For instance, RTs strongly accelerated with initial repetitions (by around 100 ms) and then asymptotically progressed (Ross, 1970). Further support for systematic learning differences comes from our observation of higher false alarm rates with increasing list length (Table 1), which—ideally—should not differ between conditions. However, when controlling for errors rates between conditions, previous studies could still demonstrate the set size effect further underlying parallel processing during retrieval of information (Wolfe, 2012; Drew and Wolfe, 2014).

Together, we can show that retrieval speed, as indicated by RTs to old/new decisions in a recognition memory task, changes as a function of set size (i.e., list length). Importantly, this relationship was both linear and logarithmic indicating serial and parallel processes, which is in line with modeling work and the notion that both bottom-up and top-down processes modulate recognition memory. Finally, the absence of age-related differences suggests that the underlying processes are unimpaired during healthy aging.

## Data Availability Statement

All data and analysis code are available from the corresponding authors upon request, including a formal project outline.

## Ethics Statement

The studies involving human participants were reviewed and approved by the Ethics committee of the University of Lübeck, Germany. The patients/participants provided their written informed consent to participate in this study.

## Author Contributions

NB and TS designed the study. JP and AS analyzed the data. NB and JP wrote the article. AS and TS provided critical revisions. All authors approved the final version of the manuscript for submission.

## Funding

This work was supported by the German Research Foundation (Deutsche Forschungsgemeinschaft Grant BU 2670/7–1 and 2670/7–2 to NB).

## Conflict of Interest

The authors declare that the research was conducted in the absence of any commercial or financial relationships that could be construed as a potential conflict of interest.

## Publisher’s Note

All claims expressed in this article are solely those of the authors and do not necessarily represent those of their affiliated organizations, or those of the publisher, the editors and the reviewers. Any product that may be evaluated in this article, or claim that may be made by its manufacturer, is not guaranteed or endorsed by the publisher.

## Footnotes

^1^ https://www.mathworks.com

^2^ https://www.ibm.com/products/spss-statistics

^3^ https://www.jamovi.org
